# Screening of Panamanian Plant Extracts for Pesticidal Properties and HPLC-Based Identification of Active Compounds

**DOI:** 10.3797/scipharm.1410-10

**Published:** 2014-12-11

**Authors:** Niels Guldbrandsen, Maria De Mieri, Mahabir Gupta, Tobias Seiser, Christine Wiebe, Joachim Dickhaut, Rüdiger Reingruber, Oliver Sorgenfrei, Matthias Hamburger

**Affiliations:** 1 Division of Pharmaceutical Biology, Department of Pharmaceutical Sciences, University of Basel, Klingelbergstrasse 50, CH-4056 Basel, Switzerland; 2 CIFLORPAN, College of Pharmacy, University of Panama, Apartado 0824-00172, Panama, Republic of Panama; 3 BASF SE, Carl-Bosch-Strasse 38, D-67056 Ludwigshafen, Germany

**Keywords:** Panamanian plant extracts, HPLC-based activity profiling, Fungicide, Insecticide, Herbicide

## Abstract

A library of 600 taxonomically diverse Panamanian plant extracts was screened for fungicidal, insecticidal, and herbicidal activities. A total of 19 active extracts were submitted to HPLC-based activity profiling, and extracts of *Bocconia frutescens*, *Miconia affinis*, *Myrcia splendens*, *Combretum aff. laxum*, and *Erythroxylum macrophyllum* were selected for the isolation of compounds. Chelerythrine (**2**), macarpine (**3**), dihydrosanguinarine (**5**), and arjunolic acid (**8**) showed moderate-to-good fungicidal activity. Myricetin-3-*O*-(6’’-*O*-galloyl)-β-galactopyranoside (**13**) showed moderate insecticidal activity, but no compound with herbicidal activity was identified.

## Introduction

Plants and their extracts have been used for a long time for crop protection. They are a promising source for pesticides due to the fact that many plants produce secondary metabolites to defend against pests. However, after evolvement of the chemical synthesis of pesticides, the importance of botanical sources decreased [1]. But still, botanical sources play an important role especially in developing countries, where there is a rich indigenous knowledge of using plants and plant extracts for crop protection [2].

Alternatively to ethnobotanical sources, the investigation of taxonomically highly diverse and unique plants has been applied successfully in drug discovery [3]. Globally, some 25 so-called biodiversity hotspots are identified combining high diversity with a high degree of endemism. The ranking is based on the number of species per 100,000 km^2^ [4]. Panama is one of the biodiversity hotspots with a highly diverse flora. Panama and its environment possess the highest diversity of plant species in the world and belong to the 25 most plant-rich countries, ranking in fourth place in the North American continent [5, 6]. Despite the small surface area, its flora comprises 9,893 vascular plant species including 1,327 (13.4%) endemic plants [7, 8]. Gupta and collaborators have shown in three reviews that the flora of Panama is extremely rich in bioactive compounds and still represents an untapped source of novel compounds for pharmaceutical, agrochemical, and cosmetic industries [9–11].

In an FP7 framework project aiming at discovering new agrochemical compounds, we screened 600 Panamanian plant extracts for fungicidal, insecticidal, and herbicidal properties. Their agrochemical potential was evaluated at BASF. A primary, highly automated screening in 96-well plates at a concentration of 2,500 ppm was done in three screens. In these assays, the fungicidal activity is tested on four pathogenic plant fungi and a ratio of the growth rate to standard is estimated by an optical density measurement. The herbicidal activity is evaluated on three plants in post- and pre-emergence, while the insecticidal activity is assessed on five different insects from four families. These screening systems are highly miniaturized and automated to provide high-throughput evaluations. Whole plants are substituted by leaf fragments and insect eggs or small larvae are used as models for real life pests. As a result, these assays are very sensitive in order to not miss any interesting activity. Follow-up tests with bigger plants in pots are then used to further characterize these initial hits to identify compounds with market potential. Selected extracts from the primary screens were submitted to a process called HPLC-based activity profiling, which combines physicochemical data recorded online with biological information in parallel to time-based HPLC fractionation [12, 13]. Most of the active constituents were isolated, characterized, and screened for pesticidal activity.

## Results and Discussion

A library of 600 extracts prepared from Panamanian plants was screened for fungicidal, insecticidal, and herbicidal activity. A total of 19 extracts fulfilled previously defined activity criteria, which were: a ratio of ≥ 0.75 for fungicidal, ≥ 50% activity against larvae and adult insects for insecticidal, and ≥ 50% (*Agrostis stolonifera* and *Poa annua*) or ≥ 80% (*Matricaria inodora*) for herbicidal activity (Tab. 1S, Supporting Information). A flow chart illustrating the further progression of samples is shown in Fig. 1. Active extracts were submitted to HPLC-based activity profiling [12, 13], and collected micro-fractions were submitted to screening in the respective assays. Based on the above activity criteria, 12 extracts were prioritized. With the aid of chromatographic and activity profiles, five extracts were then selected for a detailed investigation. Among these, two extracts were chosen for their fungicidal (Fig. 2), one extract for its insecticidal (Fig. 3), and two extracts for herbicidal activity (Fig. 4).

**Tab. S1:**
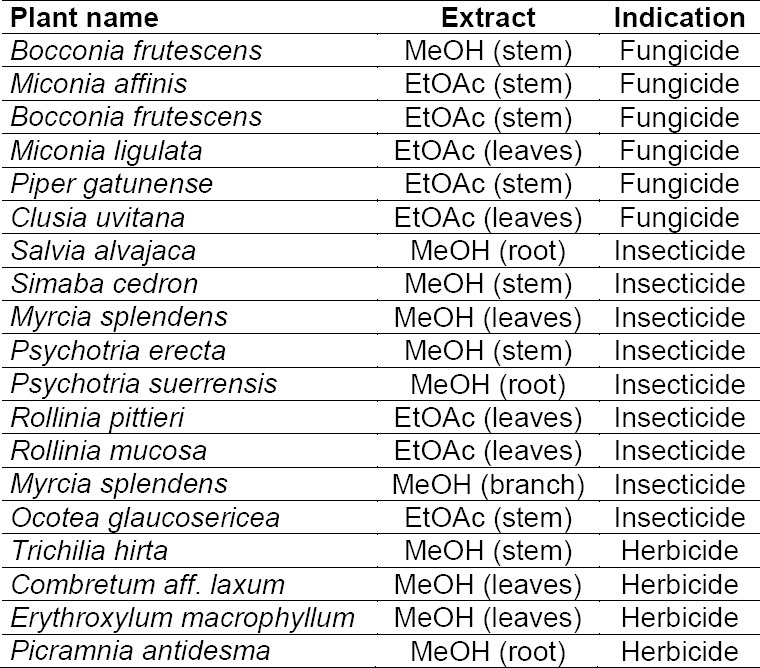
List of the 19 active extracts from the extract screening

**Fig. 1:**
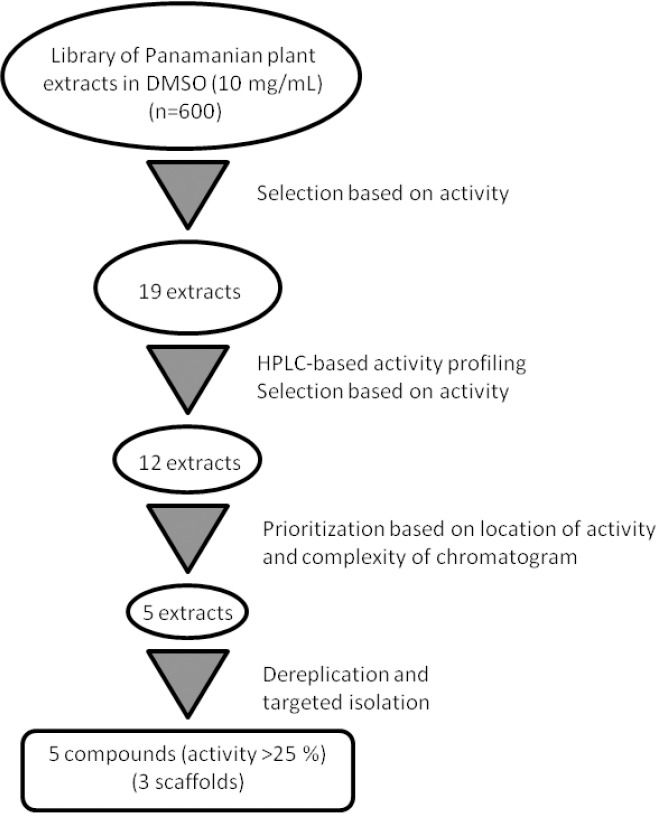
Workflow for the discovery of agrochemicals from Panamanian plant extracts

**Fig. 2:**
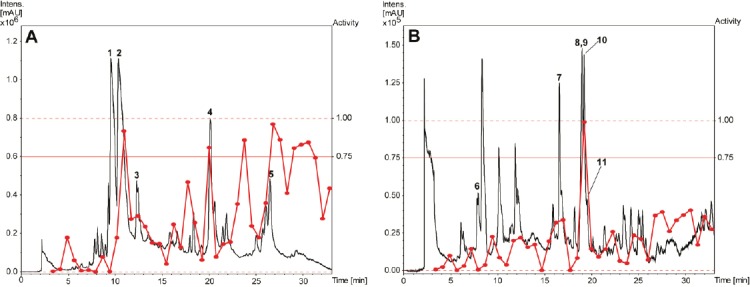
HPLC-based activity profiling of selected plant extracts for fungicidal activity against *M. oryzae*. SunFire C_18_ column (150 x 10 mm i.d., 5 μm); 5–100% MeCN/0.1% aqueous formic acid in 30 min (A), and 50-100% MeCN/0.1% aqueous formic acid in 30 min (B), 4 mL/min; detection: 200–500 nm, maxplot. (A) *Bocconia frutescens* (MeOH stem extract). (B) *Miconia affinis* (EtOAc stem extract). Activity of microfractions are shown as a red curve

**Fig. 3:**
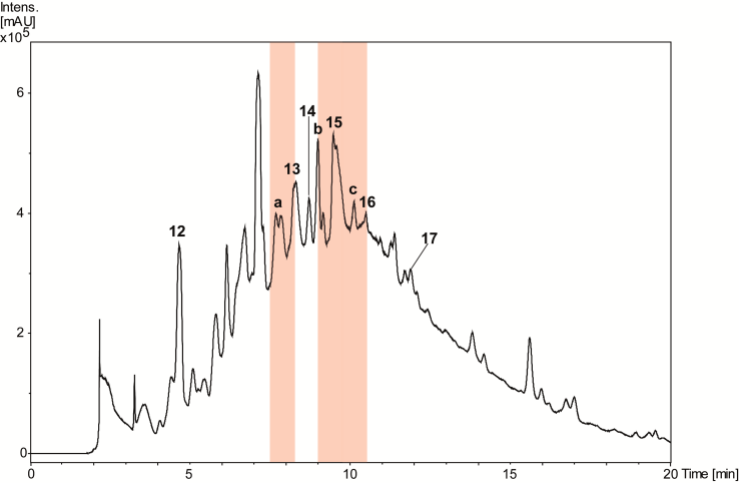
HPLC-based activity profiling of a MeOH leaf extract of *Myrcia splendens* for insecticidal activity against *Ceratitis capitata*. SunFire C_18_ column (150 x 10 mm i.d., 5 μm); 5–100% MeCN/0.1% aqueous formic acid in 30 min; 4 mL/min; time-based fractionation; detection: 200–500 nm, maxplot. Windows of insecticidal activity are highlighted in red

**Fig. 4:**
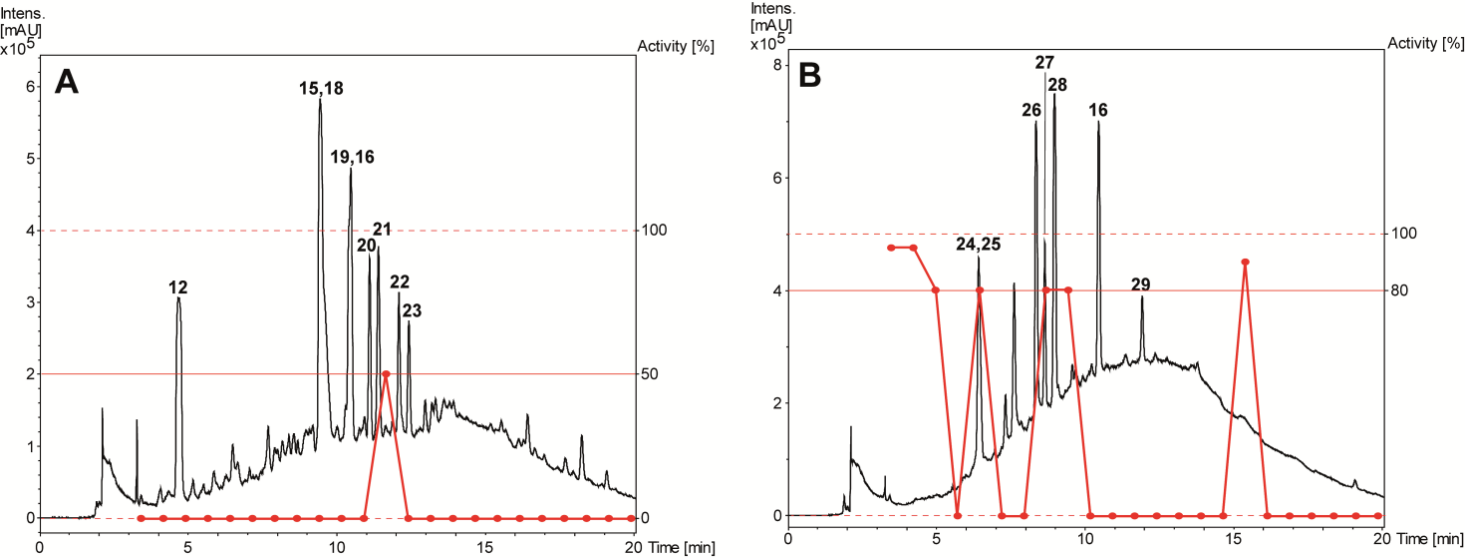
HPLC-based activity profiling of selected plant extracts for herbicidal activity. SunFire C_18_ column (150 x 10 mm i.d., 5 μm); 5–100% MeCN/0.1% aqueous formic acid in 30 min; 4 mL/min; time-based fractionation; detection: 200–500 nm, maxplot. (A) *Combretum aff. laxum* (MeOH leaf extract) against pre-emergent *Agrostis stolonifera*. (B) *Erythroxylum macrophyllum* (MeOH leaf extract) against post-emergent *Matricaria inodora*. Activity of microfractions is shown as red curves

The methanolic extract of *Bocconia frutescens* (Papaveraceae) showed fungicidal activity against *Magnaporte oryzae* in time windows corresponding to major UV-absorbing peaks (Fig. 2A). Two of the active fractions and one additional fraction also showed activity against other fungal strains (Fig 1S, Supporting Information). The two early-eluting main peaks were identified as sanguinarine (**1**) [14] and chelerythrine (**2**) [14] (Fig. 5). Compound **2** showed moderate activity against *Botryotinia fuckeliana*, *M. oryzae*, *Phytophtora infestans*, and *Septoria tritici*. The late-eluting active peaks were identified as oxysanguinarine (**4**) [15] and dihydrosanguinarine (**5**) [14]. Compound **4** showed no fungicidal activity, while **5** was active against *M. oryzae, P. infestans, and S. tritici*. Macarpine (**3**) [16] was in a microfraction active against *P. infestans* (Fig 1SB, Supporting Information). The purified compound showed good fungicidal activity against *P. infestans* and *M. oryzae*. With the exception of **4**, the compounds had been previously reported from *B. frutescens* [17, 18].

**Fig. 1S:**
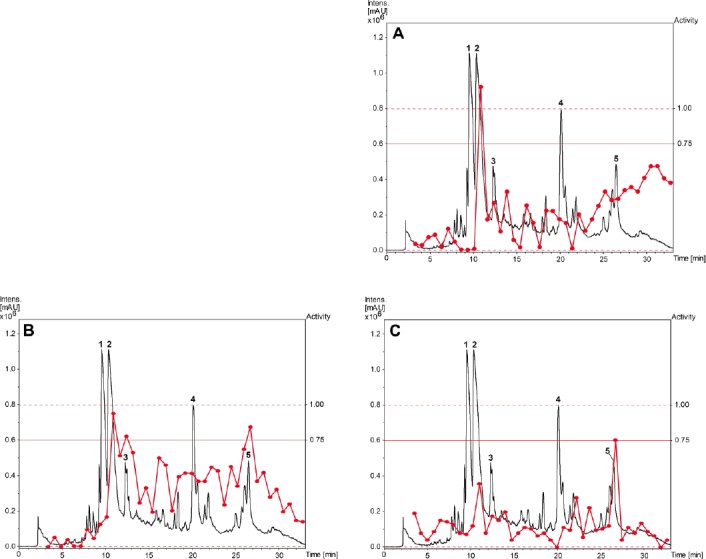
Profiles of the MeOH stem extracts of Bocconia frutescens for the plant pathogenic fungi Botryotinia fuckeliana (A), Phytophthora infestans (B), and Septoria tritici (C)

**Fig. 5:**
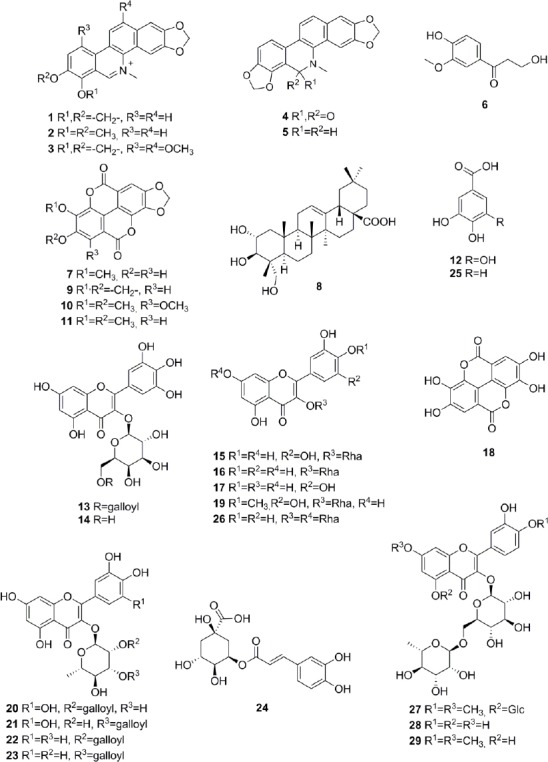
Structures of identified compounds: sanguinarine (**1**), chelerythrine (**2**), macarpine (**3**), oxysanguinarine (**4**), dihydrosanguinarine (**5**), β-hydroxypropiovanillone (**6**), 3’-*O*-methyl-3,4-*O*,*O*-methyleneellagic acid (**7**), arjunolic acid (**8**), 3,4:3’,4’-bis(*O*-*O*-methylene)ellagic acid (**9**), 3’,4’,5’-tri-*O*-methyl-3,4-*O*,*O*-methyleneflavellagic acid (**10**), 3’4’-di-*O*-methyl-3,4-*O*,*O*-methyleneellagic acid (**11**), gallic acid (**12**), myricetin-3-*O*-(6’’-*O*-galloyl)-β-galactopyranoside (**13**), myricetin-3-*O*-β-galactopyranoside (**14**), myricitrin (**15**), quercitrin (**16**), myricetin (**17**), ellagic acid (**18**), mearnsitrin (**19**), 2’’-*O*-galloylmyricitrin (**20**), 3’’-*O*-galloylmyricitrin (**21**), 2’’-*O*-galloylquercitrin (**22**), 3’’-*O*-galloylquercitrin (**23**), neochlorogenic acid (**24**), protocatechuic acid (**25**), quercitrin-7-*O*-α-rhamnopyranoside (**26**), 5-*O*-β-glucopyranosylombuin-3-*O*-β-rutinoside (**27**), rutin (**28**), and ombuin-3-*O*-β-rutinoside (**29**)

The profile of the ethyl acetate extract of *Miconia affinis* (Melastomataceae) showed one fraction active against *M. oryzae* (Fig. 2B) and *S. tritici*. This fraction consisted of three strongly UV-absorbing peaks (**9**–**11**) and one non-UV active compound (**8**) (Fig. 5). Peak **10** was purified and identified as 3’,4’,5’-tri-*O*-methyl-3,4-*O*,*O*-methyleneflavellagic acid [19]. UV and MS data of the other two UV-absorbing peaks were indicative of 3,4:3’,4’-bis(*O*-*O*-methylene)ellagic acid (**9**) [20] and 3’,4’-di-*O*-methyl-3,4-*O*,*O*-methyleneellagic acid (**11**) [19], and were not further pursued. Arjunolic acid (**8**) [21] was purified by normal phase flash chromatography, and its presence in the active fraction was confirmed by HPLC-DAD-ELSD. Compound **8** was active against *M. oryzae* and *S. tritici*. In previous studies [22, 23], the fungicidal activity of arjunolic acid (**8**) in a mixture with asiatic acid was reported, while in the current study the activity of purified **8** was confirmed. Two additional compounds outside of the active time window were also isolated and identified as β-hydroxypropiovanillone (**6**) [24] and 3’-*O*-methyl-3,4-*O*,*O*-methyleneellagic acid (**7**) [25]. All compounds are reported for the first time from *M. affinis*, since no phytochemical studies have been conducted on this species before.

A broad hump in the chromatogram of the methanolic extract of *Myrcia splendens* (Myrtaceae) indicated the presence of tannins (Fig. 3). However, two distinct windows of insecticidal activity against *Ceratitis capitata* were seen between t_R_ 7–10 min. After large-scale extraction, peaks **a** and **b** depleted, and **c** even disappeared, while peak **15** was extremely enriched in the crude extract. Prior to HPLC purification, the extract was separated over polyamide yielding five tannin-depleted fractions (Fig 2S, Supporting Information). From the first active time-window, compound **13** was isolated and identified as myricetin-3-*O*-(6’’-*O*-galloyl)-β-galactopyranoside [26] (Fig. 5). The compound showed weak activity against *C. capitata* at 2500 ppm. From the second active time window, inactive myricitrin (**15**) [27] and quercitrin (**16**) [28] were isolated. Additional compounds isolated from fractions outside of the active time windows were gallic acid (**12**), myricetin-3-*O*-β-galactopyranoside (**14**) [29], and myricetin (**17**) [30]. Compound **15** had been previously reported from *M. splendens* [31], while the other compounds were new for the species.

**Fig. 2S:**
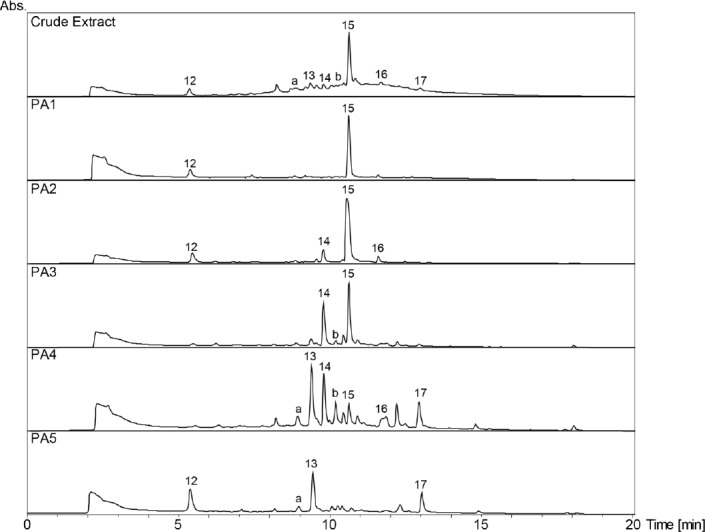
HPLC-DAD chromatograms of the crude extract and its polyamide fractions (PA1–PA5) of Myrcia splendens. SunFire C18 column (150 x 3 mm i.d., 3.5 μm); 5–100% MeCN/0.1% aqueous formic acid in 30min, 0.4 mL/min; detection: 210–700nm, maxplot.

The methanolic leaf extract of *Combretum affinis laxum* (Combretaceae) showed herbicidal activity against pre-emergent *Agrostis stolonifera* (Fig 4A), and post-emergent *Poa annua* in the time range of peak **21**. Tannins in the extract were removed by filtration over polyamide, and 2’’-*O*-galloylmyricitrin (**20**) [32], 3’’-*O*-galloylmyricitrin (**21**) [32], 2’’-*O*-galloylquercitrin (**22**) [33], and 3’’-*O*-galloylquercitrin (**23**) [34, 35] were isolated by HPLC from fractions PA4 and PA5 (Fig 3S, Supporting Information). Compound **21** showed no significant herbicidal activity. Ellagic acid (**18**) was obtained from PA5 and confirmed by spiking with a commercial sample. In addition, inactive compounds **12**, **15**, **16** were isolated, together with mearnsitrin (**19**) [36]. All compounds were new for *C. aff. laxum*, since no phytochemical data have been previously reported on this species.

**Fig. 3S:**
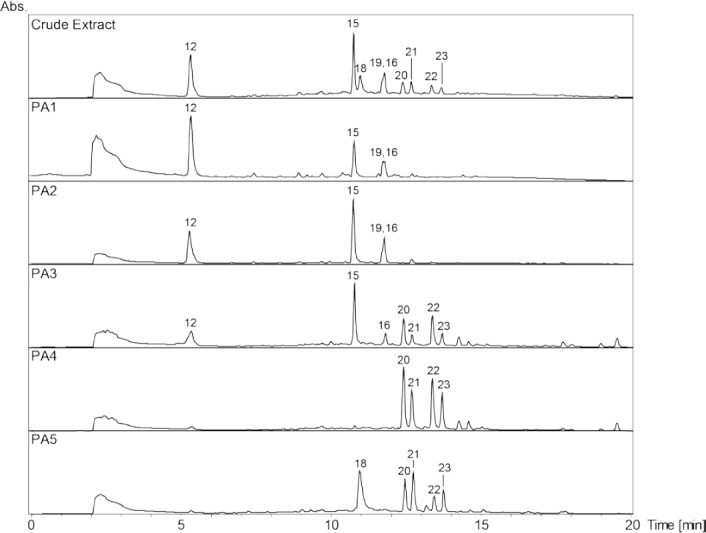
HPLC-DAD chromatograms of the crude extract and its polyamide fractions (PA1–PA5) of Combretum aff. laxum. SunFire C18 column (150 x 3 mm i.d., 3.5 μm); 5–100% MeCN/0.1% aqueous formic acid in 30min, 0.4 mL/min; detection: 210–700nm, maxplot.

The extract of *Erythroxylum macrophyllum* (Erythroxylaceae) showed distinct activity against post-emergent *M. inodora*, even though the broad hump in the HPLC chromatogram was indicative of tannins (Fig 4B). In time windows t_R_ 3-5 min and t_R_ 15–16 min, the activity could not be correlated to a peak in the UV or MS traces. The extract was filtered over polyamide, and five tannin-depleted fractions were obtained (Fig 4S, Supporting Information). Compounds in the active time windows were purified by HPLC, and identified as neochlorogenic acid (**24**) [37, 38], protocatechuic acid (**25**) [39], quercetin-3,7-*O*-α-dirhamnopyranoside (**26**) [40], 5-*O*-β-glucopyranosylombuin-3-*O*-β-rutinoside (**27**) [41], and rutin (**28**) [42]. However, none of the flavonoids showed activity in the herbicidal assay when tested as pure compounds. In addition, **16** and ombuin-3-*O*-β-rutinoside (**29**) [41] were isolated. All compounds are reported here for the first time from *E. macrophyllum*.

**Fig. 4S:**
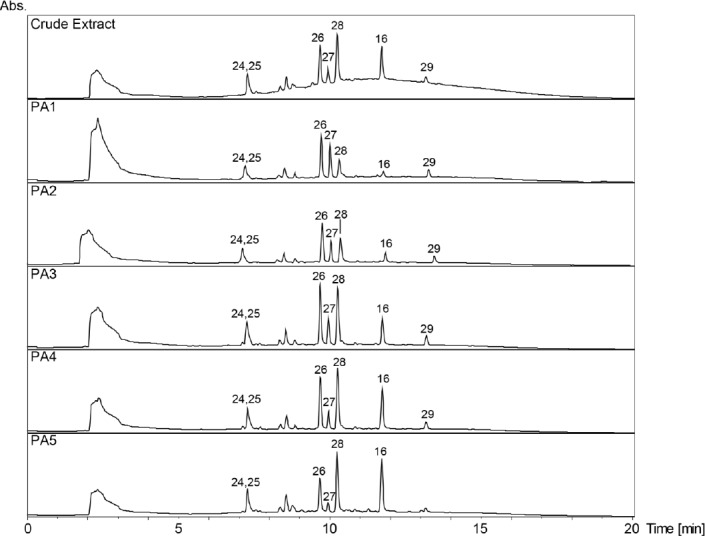
HPLC-DAD chromatograms of the crude extract and its polyamide fractions (PA1–PA5) of Erythroxylum macrophyllum. SunFire C18 column (150 x 3 mm i.d., 3.5 μm); 5–100% MeCN/0.1% aqueous formic acid in 30min, 0.4 mL/min; detection: 210–700nm, maxplot.

In total, four fungicidal and one weakly insecticidal natural product were discovered by means of HPLC-based activity profiling. In contrast, none of the compounds purified from active time windows of *C. aff. laxum* and *E. macrophyllum* showed herbicidal activity. The activity in these time windows may have been, at least in part, due to the presence of tannins. This might have been confirmed by a retest for activity of tannin-depleted extracts. The example of fungicidal compounds showed that the profiling approach could be efficiently used for discovery of bioactive compounds of possible agrochemical interest.

**Tab. 1:**
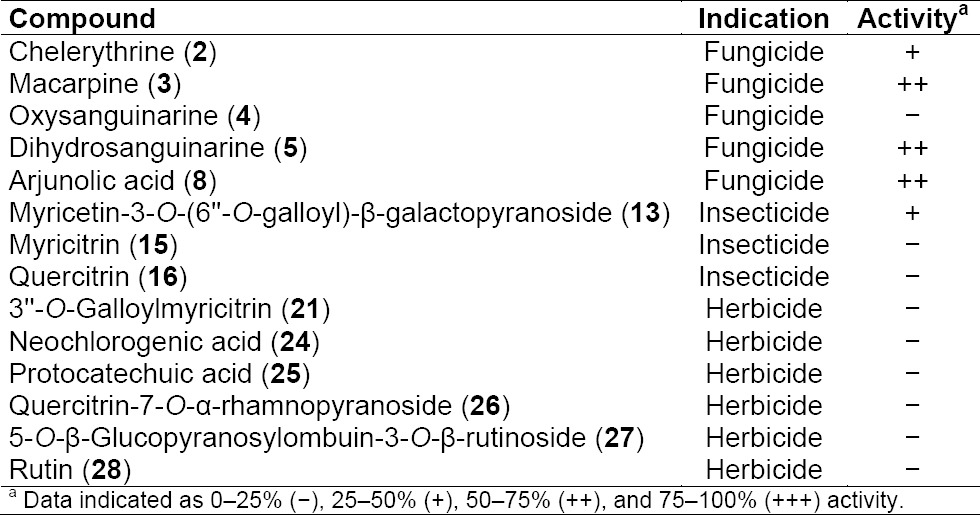
Activity of isolated and tested compounds

## Experimental

### General Experimental Procedures

Quercitrin (**16**, ≥98%) and polyamide (particle size: 0.05-0.16 mm) were purchased from Carl Roth. Rutin (**27**, ≥94%) was from Sigma-Aldrich. HPLC-grade acetonitrile and methanol (Reuss Chemie AG), and distilled water were used for HPLC separations. Preparative HPLC was carried out on an LC 8A preparative liquid chromatograph equipped with an SPD-M10A VP PDA detector (all Shimadzu). A SunFire C_18_ column (150 x 30 mm i.d., 5 μm; Waters) connected to a pre-column (10 x 30 mm) was used, at a flow rate of 20 mL/min. HPLC-based activity profiling was performed on an Agilent 1100 system equipped with a PDA detector. A SunFire C_18_ column (150 x 10 mm i.d., 5 μm; Waters) connected to a pre-column (10 x 10 mm) was used, at a flow rate of 4 mL/min. Time-based fractions were collected with a Gilson FC204 fraction collector. Analytical HPLC-DAD-ELSD chromatography was performed on a Waters 2690 Alliance system equipped with a 996 PDA detector and an Alltech ELSD 2000ES. A SunFire C_18_ column (150 x 3 mm i.d., 3.5 μm; Waters) connected to a pre-column (10 x 3 mm) was used, at a flow rate of 0.4 mL/min. Silica gel flash chromatography was performed on an Interchim Puri Flash 4100 system. ESI-MS spectra were obtained on an Esquire 3000 Plus ion trap mass spectrometer (Bruker Daltonics). ESI-TOF-MS spectra were recorded in positive mode on a Bruker microTOF ESI-MS system. Mass calibration was done with a reference solution of 0.1% sodium formate in 2-propanol/water (1:1) containing 5 mM NaOH. NMR spectra were recorded on an Avance III 500 MHz spectrometer (Bruker BioSpin) equipped with a 1-mm TXI microprobe and a 5-mm BBO probe.

### Plant Material

Stems of *Bocconia frutescens L*. were collected in August 2012 in El Valle de Antón, La Mesa, Coclé, Panama. Stems of *Miconia affinis DC*. were collected in October 2007 in Parque Nacional Chagres, section Cerro Azul, Panama. Leaves of *Myrcia splendens (SW.) DC*. were collected in June 2012 in Parque Nacional Altos de Campana, Panama. Stems of *Combretum affinis laxum Jacq*. were collected in November 1996 in Punta Muñiz, Parque Nacional Coiba, Panama. Leaves of *Erythroxylum macrophyllum Kunth* were collected in January 1993 in Parque Nacional Altos de Campana, Panama. The plant material was identified by Alex Espinosa, and voucher specimens have been deposited at the Herbarium of the University of Panama (PMA). Also, vouchers are kept at the Division of Pharmaceutical Biology, University of Basel: Nr. 844 (*B. frutescens*), Nr. 845 (*M. affinis*), Nr. 901 (*M. splendens*), Nr. 866 (*C. aff. laxum*), and Nr. 867 (*E. lucidum*).

### HPLC-Based Activity Profiling

Extracts dissolved in DMSO (50 mg/mL) were separated by semi-preparative HPLC. Two aliquots of 200 μL corresponding to 10 mg extract were injected. For two ethyl acetate extracts (stems of *Bocconia frutescens* and leaves of *Clusia uvitana*), a gradient of 50–100% MeCN in 30 min in 0.1% aqueous formic acid was used. For the other extracts, a gradient of 5-100% MeCN in 30 min in 0.1% aqueous formic acid was employed. Fractions of 0.75 min were collected from t = 3 min to t = 33 min. Fractions were transferred into 96-deepwell plates, evaporated, and submitted to screening.

### Extraction and Isolation

Powdered stems of *B. frutescens* (1,002.0 g) were percolated with 15 L MeOH to afford 45.9 g of extract. A portion (10.0 g) of the extract was submitted to silica gel flash chromatography using CH_2_Cl_2_ for 30 min, followed by a gradient of 0–5% in 60 min, 5% over 30 min, 5–10% in 30 min, and 10% MeOH in CH_2_Cl_2_ over 60 min, at a flow rate of 50 mL/min. Eight fractions (Fr. 1-8) were combined on the basis of TLC patterns. Fr. 1 (409.3 mg) was separated by preparative HPLC (80% aqueous MeCN with 0.1% formic acid) to afford dihydrosanguinarine (**5**, 231.7 mg, t_R_ 11.4 min). A portion (501.9 mg) of Fr. 7 (1620.1 mg) was separated by flash chromatography on silica gel using hexane (solvent A) and ethyl acetate (solvent B) at a flow rate of 10 mL/min. A gradient of 0-19% B in 19 min, 19% B over 5 min, 19–40% B in 21 min, and 40–100% B in 41 min, followed by 100% B over 81 min yielded six fractions (Fr. 7a–7f). Colorless crystals precipitated from Fr. 7d-7f, and were recrystallized from ethyl acetate/CH_2_Cl_2_ to afford oxysanguinarine (**4**, 18.6 mg). Fractions 7b–7d were submitted to preparative HPLC (aqueous MeCN with 0.025% TFA). Sanguinarine (**1**, 67.0 mg, t_R_ 8.1 min) was obtained from Fr. 7b (123.3 mg) using 32% MeCN. For Fr. 7c (163.4 mg) and 7d (123.2 mg), 35% MeCN was used to afford chelerythrine (**2**, 105.1 mg, t_R_ 7.6 min) and macarpine (**3**, 9.5 mg, t_R_ 13.6 min).

Powdered stems of *M. affinis* (1,001.2 g) were percolated with ethyl acetate (11 L) to afford 4.5 g of extract. A portion (2.9 g) of the extract was submitted to silica gel flash chromatography using CH_2_Cl_2_ (solvent A) and ethyl acetate (solvent B). A gradient of 0–100% B in 120 min, followed by 100% B over 30 min was used, at a flow rate of 40 mL/min to afford 12 fractions (Fr. 1–12). Fr. 5 (370.1 mg) was separated by preparative HPLC using 45% aqueous MeCN to give 3’-*O*-methyl-3,4-*O*,*O*-methyleneellagic acid (**7**, 1.8 mg, t_R_ 12.8 min), 3’,4’,5’-tri-*O*-methyl-3,4-*O*,*O*-methyleneflavellagic acid (**10**, 4.2 mg, t_R_ 23.9 min), and a mixture of 3’4’-di-*O*-methyl-3,4-*O*,*O*-methyleneellagic acid (**11**, t_R_ 24.9 min) and **10**. From Fr. 8 (102.5 mg), a mixture containing 3,4:3’,4’-bis(*O*,*O*-methylene)ellagic acid (**9**) precipitated after dissolution in DMSO. The supernatant of Fr. 8 was submitted to preparative HPLC (15% aqueous MeCN) to afford β-hydroxypropiovanillone (**6**, 1.4 mg, t_R_ 12.9 min). Fr. 12 (396.7 mg) was separated by flash chromatography on silica gel, using CH_2_Cl_2_ (solvent A) and MeOH (solvent B) as the mobile phase. A gradient of 0–6% B in 8 min, 6% B over 22 min, and 6–100% B in 10 min afforded arjunolic acid (**8**, 241.1 mg).

Powdered leaves of *M. splendens* (801.8 g) were percolated with MeOH (12 L) to afford 217.0 g of extract. A portion (10.2 g) of the extract was redissolved in 200 mL MeOH and separated on a polyamide column (50-160 μm, 200 g; Roth) with MeOH as eluent. Three fractions (PA1–PA3) of 1 L each, one fraction (PA4) of 3 L, and one fraction (PA5) of 5 L were collected. Fraction PA2 (682.4 mg) was separated by preparative HPLC using 25% aqueous MeCN to afford myricitrin (**15**, t_R_ 7.0 min) and quercitrin (**16**, 5.0 mg, t_R_ 10.2 min). Final purification of **15** was achieved with 20% aqueous MeCN (134.5 mg, 13.7 min). Preparative HPLC of fraction PA3 (19% aqueous MeCN) yielded myricetin-3-*O*-β-galactopyranoside (**14**, t_R_ 10.2 min) and **15** (7.8 mg, t_R_ 15.9 min). **14** was finally purified by semi-preparative HPLC using 17% MeCN in 0.05% aqueous formic acid (4.2 mg, t_R_ 7.6 min). Fraction PA5 was separated by preparative HPLC with a gradient of MeCN in 0.05% aqueous formic acid (5–40% over 15 min). Gallic acid (**12**, 8.9 mg, t_R_ 7.6 min), myricetin-3-*O*-(6’’-*O*-galloyl)-β-galactopyranoside (**13**, t_R_ 12.2 min), and myricetin (**17**, 4.1 mg, t_R_ 17.3 min) were obtained. Final purification of **13** by semi-preparative HPLC was with 15% MeCN in 0.05% aqueous formic acid (6.9 mg, t_R_ 9.5 min).

Powdered leaves of *C. aff. laxum* (197.7 g) were percolated with MeOH (5 L) to afford 13.2 g of extract. A portion (10.1 g) of the extract was redissolved in 200 mL MeOH and submitted to polyamide (200 g) filtration. Four fractions (PA1-PA4) of 1 L each, and one fraction of 3 L (PA5) were collected. Fractions PA2, PA4, and PA5 were submitted to preparative HPLC. A portion (500.0 mg) of fraction PA2 (1036.4 mg) was separated with 25% MeCN in 0.05% aqueous formic acid to afford myricitrin (**15**, 54.0 mg, t_R_ 6.8 min), mearnsitrin (**19**, 0.64 mg, t_R_ 9.5 min), and quercitrin (**16**, 8.1 mg, 9.8 min). Fraction PA4 (160.7 mg) was separated with 30% MeCN in 0.05% aqueous formic acid to give 2”-*O*-galloylmyricitrin (**20**, 10.5 mg, t_R_ 7.4 min), 3”-*O*-galloylmyricitrin (**21**, 5.3 mg, t_R_ 8.3 min), 2”-*O*-galloylquercitrin (**22**, 6.9 mg, t_R_ 10.5 min), and 3”-*O*-galloylquercitrin (**23**, 5.0 mg, 11.9 min). Fraction PA5 (785.6 mg) was separated with a gradient of 21–41% MeCN in 0.05% aqueous formic acid over 30 min to afford ellagic acid (**18**, 1.3 mg, t_R_ 9.5 min), **20** (10.0 mg, t_R_ 14.3 min), **21** (12.6 mg, 15.2 min), **22** (4.9 mg, 18.0 min), and **23** (6.0 mg, 19.1 min).

Powdered leaves of *E. lucidum* (601.5 g) were percolated with MeOH (11 L) to give 77.3 g of extract. A portion (20.3 g) of the extract was redissolved in 200 mL MeOH and submitted to polyamide (200 g) filtration. Four fractions (PA1-PA4) of 250 mL each, and one fraction (PA5) of 5 L were collected. A portion (700.1 mg) of fraction PA3 (2,183.2 mg) was submitted to preparative HPLC (gradient of 10-55% MeCN in 0.05% aqueous formic acid over 20 min) to afford quercitrin-7-O-α-rhamnopyranoside (**26**, 20.1 mg, t_R_ 10.2 min), rutin (**28**, 22.9 mg, t_R_ 11.0 min), quercitrin (**16**, 13.1 mg, t_R_ 13.2 min), and ombuin-3-*O*-β-rutinoside (**29**, 6.6 mg, t_R_ 15.1 min). Two mixed fractions (t_R_ 8.4 min and t_R_ 10.5 min) were submitted to final purification by preparative HPLC to afford neochlorogenic acid (**24**, 6.2 mg, t_R_ 5.6 min), protocatechuic acid (**25**, 2.0 mg, t_R_ 6.2 min), **26** (2.1 mg, t_R_ 6.5 min), and 5-*O*-β-glucopyranosylombuin-3-*O*-β-rutinoside (**27**, 8.2 mg, t_R_ 7.5 min), respectively.

Compounds were identified with the aid of ^1^H- and 2D-NMR, and ESI-MS spectroscopy, and by comparison with literature data. The purity of isolated compounds was >95% as determined by NMR, except for compounds **3** (90%), **4** (80%), **25** (90%), and **29** (80%).

### Fungicidal Assay

The activity against phytopathogenic fungi (*Botryotinia fuckeliana*, *Magnaporthe oryzae*, *Phytophtora infestans*, and *Septoria tritici*) could be demonstrated by the treatment of fungal spore suspensions and analysis of the growth in microplates using a robot system.

The tests were done in 96-well microtiter plates. Compounds were transferred as DMSO solutions into empty plates, followed by the addition of a spore suspension of the fungus of interest in a nutrient solution. Compounds were tested either in a single concentration, or as serial dilutions at 10 concentrations. Each plate contained eight solvent control wells and eight reference wells containing a known fungicide. The plates were incubated at 23°C and 90% relative humidity. Fungal growth was assessed by measuring the optical density at 620 nm, immediately after treatment, and 10 times in intervals of 15 hours. In order to calculate the activity of a compound at a given concentration, the optical density values of each measurement of a compound was compared with those of the control and the reference, giving results from 0 to 1, whereby higher values indicated higher activity. ED_50_ values were calculated with the aid of the dilution series. A compound having an activity ratio ≥ 0.75, or an ED_50_ ≤ 10 mg/l was considered as active.

### Insecticidal Assay

Tested insect species were *Anthonomus grandis*, *Heliothis virescens*, *Ceratitis capitata*, *Megoura viciae*, and *Myzus persicae*. Insecticidal activity, either as a contact or systemic insecticide, against piercing/sucking insects (adults and offspring) was assessed in a test unit consisting of 24-well microtiter plates containing broad bean leaf disks. The compounds were formulated using a solution containing 75% v/v water and 25% v/v DMSO. Different concentrations of formulated compounds were sprayed onto the leaf disks at 2.5 µl, using a custom-built micro-atomizer. Two replicates were prepared. After application, leaf disks were air-dried, and 5–8 adult insects were placed onto the leaf disks placed into wells of a microtiter plate. Insects were then allowed to suck on the treated leaf disks, and were incubated at about 23 ± 1°C and about 50 ± 5% relative humidity for 5 days. Mortality was visually assessed.

Activity against biting insects (larvae) was evaluated in a test unit consisting of 24-well microtiter plates containing an insect diet and 20-30 insect eggs. Test compounds were formulated using a solution containing 75% v/v water and 25% v/v DMSO. Aliquots (20 μl) of different concentrations of formulated compounds were sprayed onto the insect diet using a custom-built micro-atomizer. Two replicates were used. After application, microtiter plates were incubated for 5 days at 23 ± 1°C and 50 ± 5% relative humidity. Egg and larval mortality was then visually assessed. Compounds with ≥ 50% mortality in adult insects and larvae were considered as active.

### Herbicidal Assay

Herbicidal activity was assessed on pre- and post-emergent *Matricaria inodora*, *Agrostis stolonifera*, and *Poa annua*. The culture containers used were plastic 96-well plates containing peat substrate. For the post-emergence treatment, the test plants, once they reached a height of 1-3 cm (depending on the plant species), were sprayed via a spray nozzle with the test compounds in 1,000 ppm DMSO solution. The application rate corresponded to 2 kg/ha, with an application volume of 200 L/ha. Plants were kept at 20–35°C. The test period extended over 7 days. During this time, the plants were tended, and their response to the individual treatments was evaluated visually. The cutoff for herbicidal activity was ≥ 50% inhibition of growth (or 80% in the case of *Matricaria inodora*) of the treated weed, either pre- or post-emergence.

**Tab. 2S:**
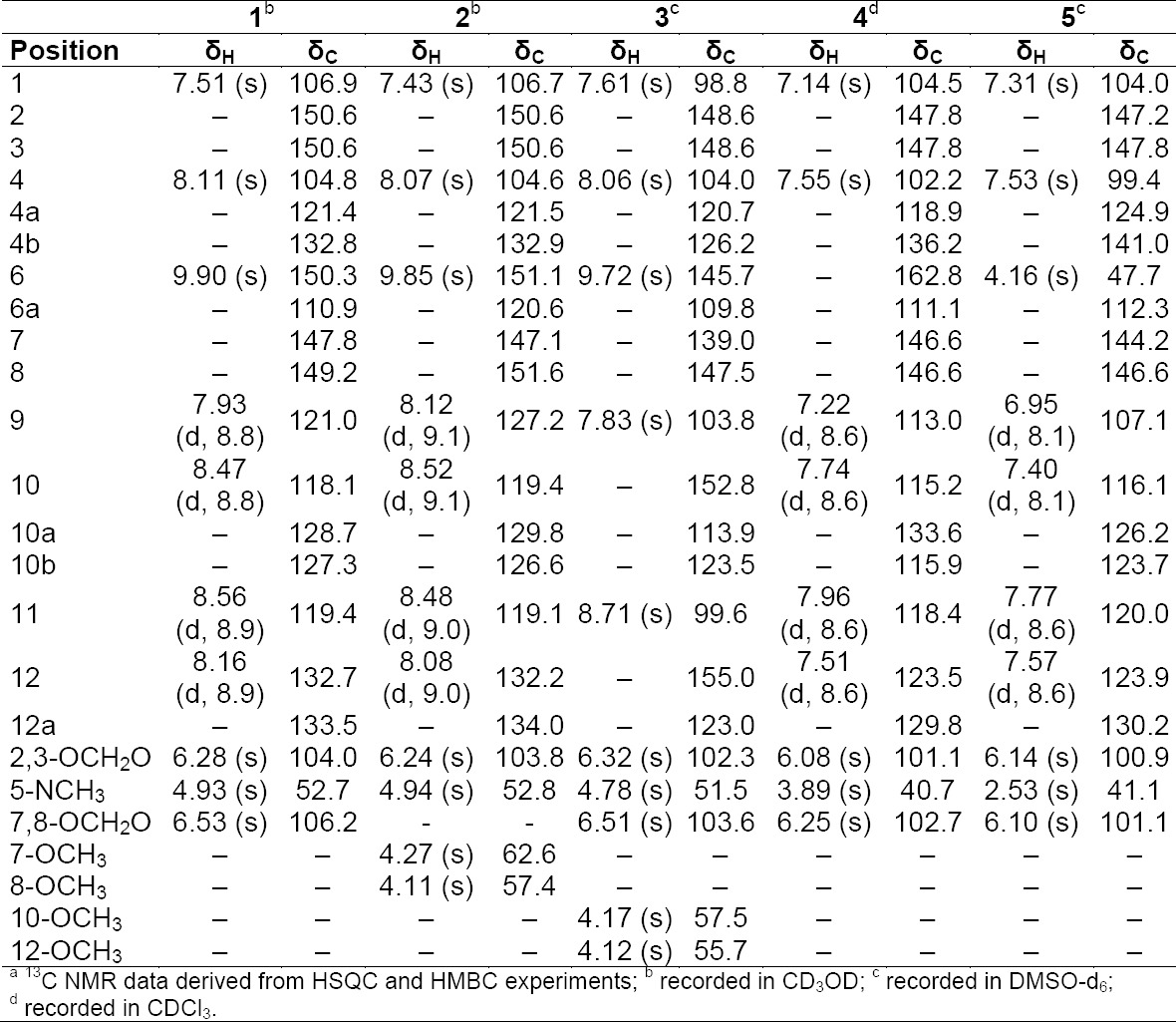
^1^H and ^13^C NMR data (500 MHz) of compounds **1–5**

**Tab. 3S:**
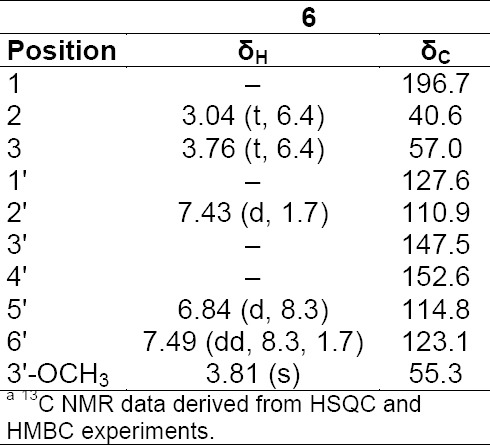
^1^H and ^13^C NMR data (500 MHz) of compound **6** in DMSO-d6

**Tab. 4S:**
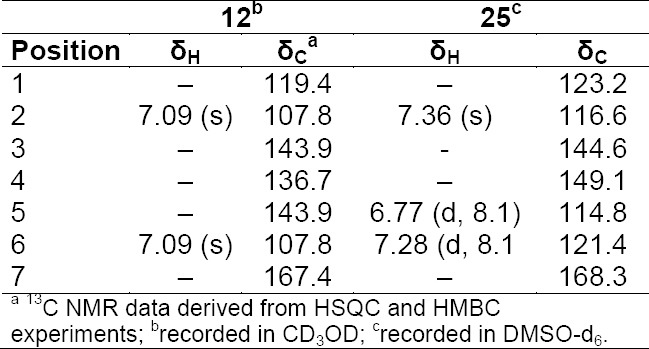
^1^H and ^13^C NMR data (500 MHz) of compounds **12** and **25**

**Tab. 5S:**
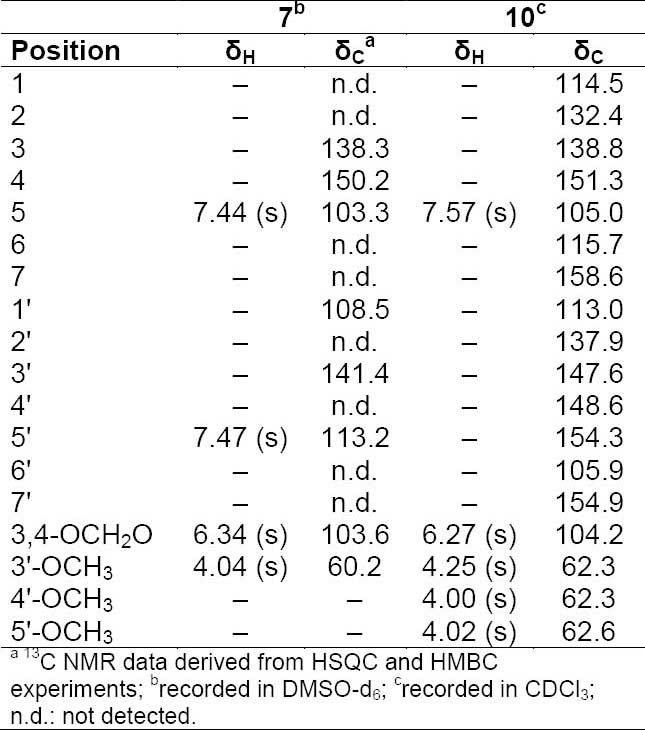
^1^H and ^13^C NMR (500 MHz) of compound **7** and **10**

**Tab. 6S:**
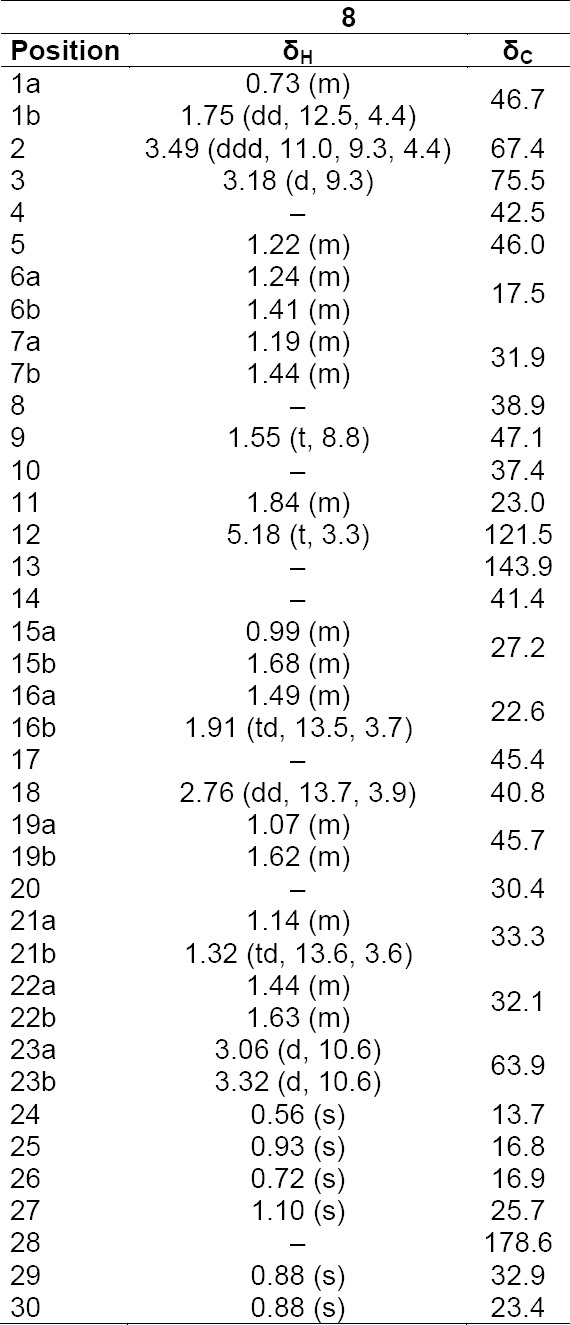
^1^H and ^13^C NMR data (500 MHz) of compound **8** in DMSO-d6

**Tab. 7S:**
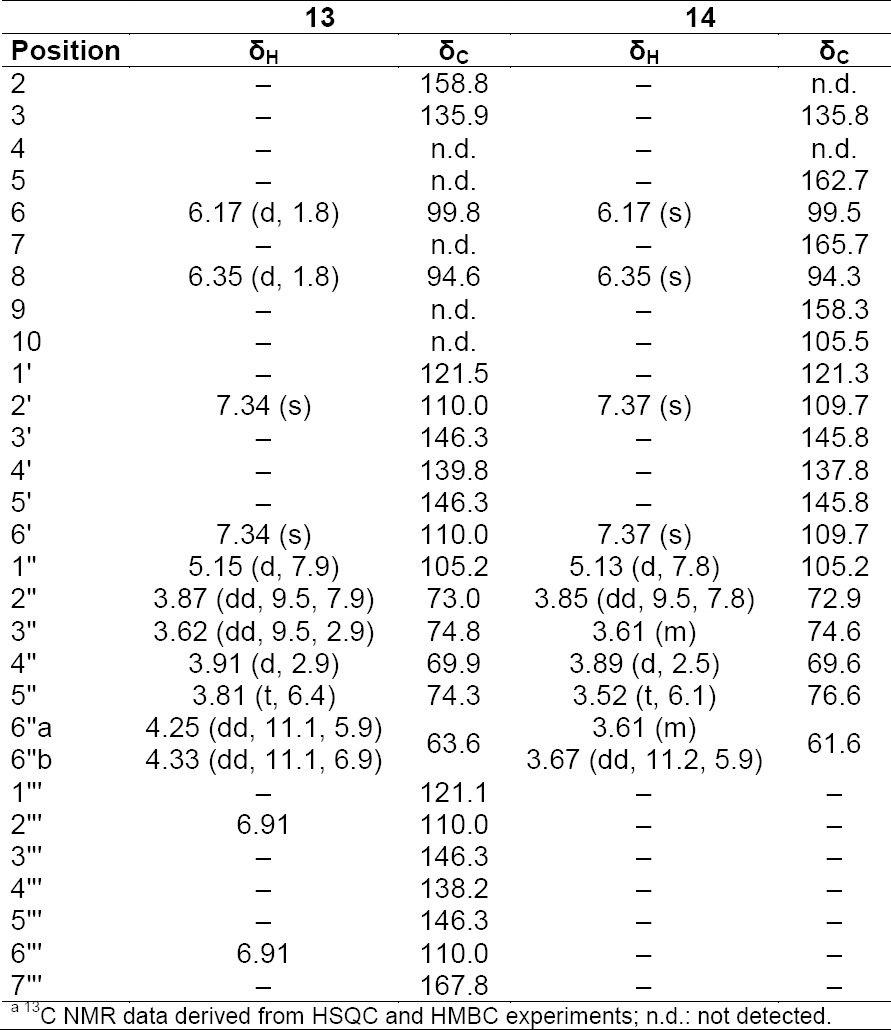
^1^H and ^13^C NMR (500 MHz) of compounds **13** and **14** in CD3OD

**Tab. 8S:**
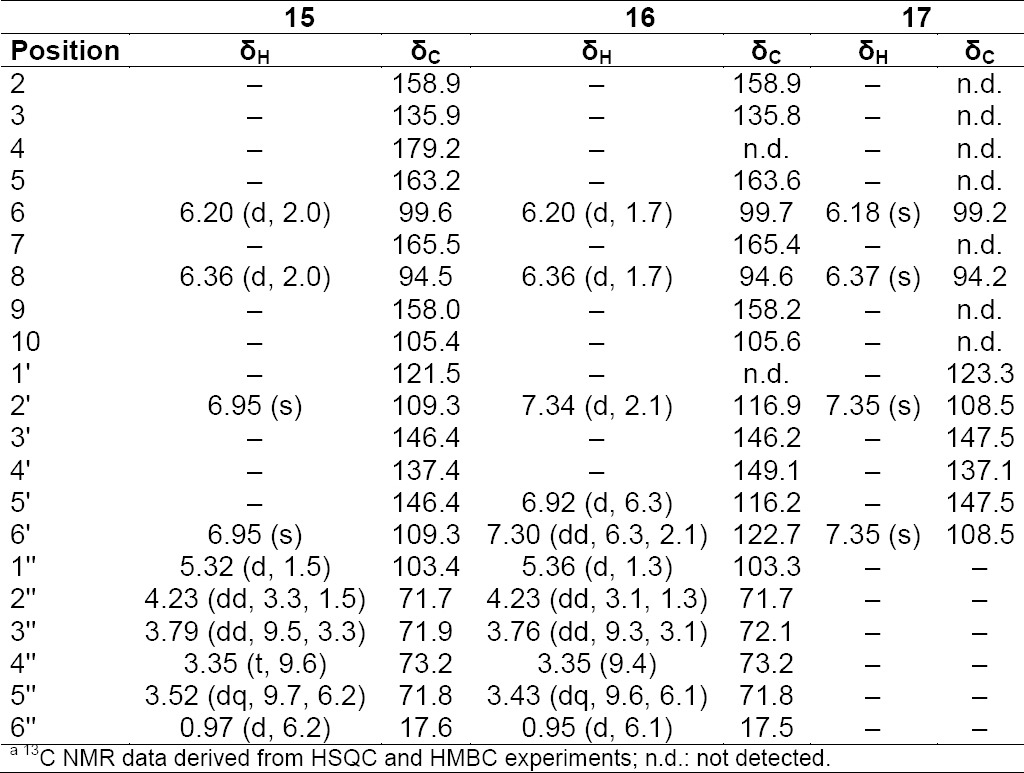
^1^H and ^13^C NMR (500 MHz) of compounds **15–17** in CD3OD

**Tab. 9S:**
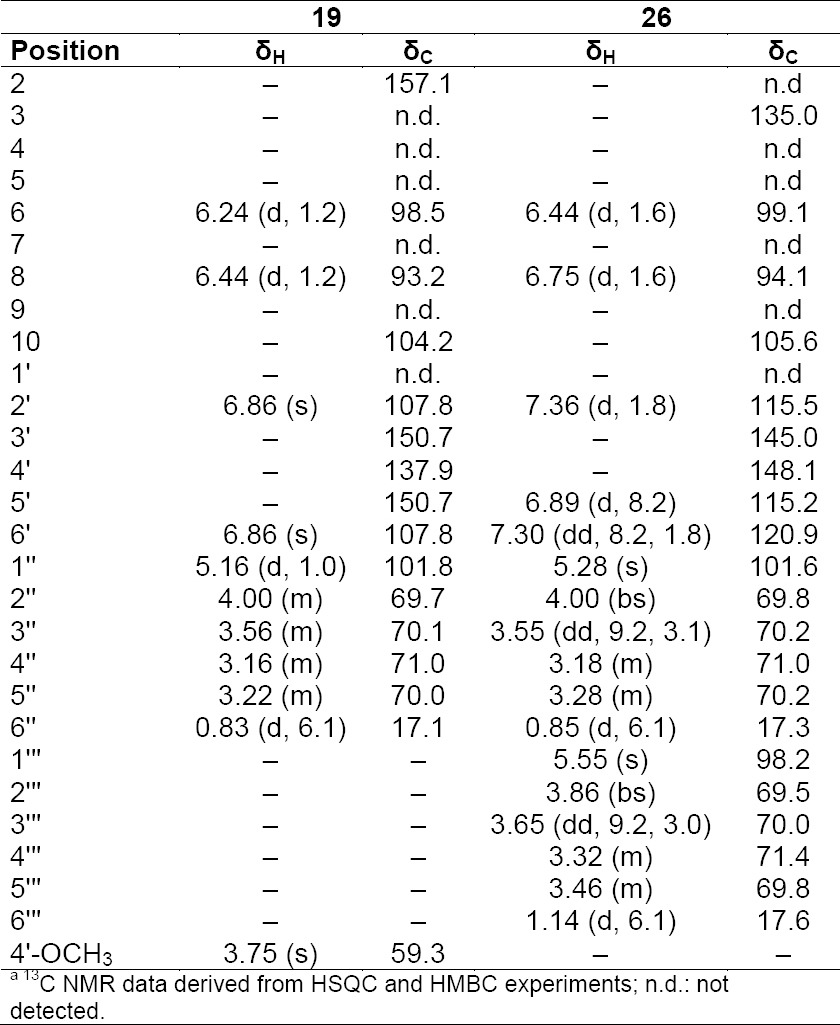
^1^H and ^13^C NMR (500 MHz) of compounds **19** and **26** in DMSO-d6

**Tab. 10S:**
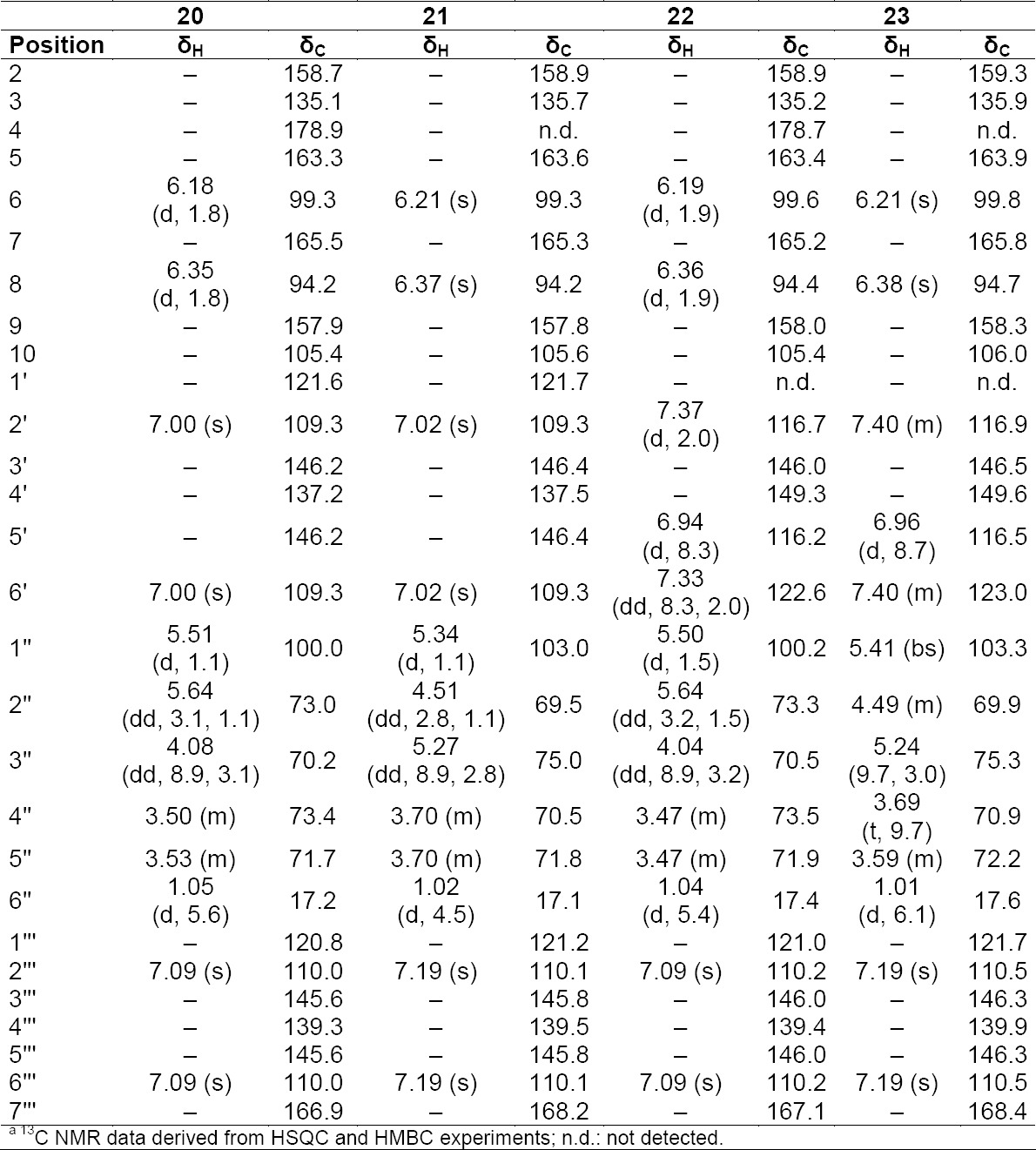
^1^H and ^13^C NMR (500 MHz) of compounds **20–23** in CD3OD

**Tab. 11S:**
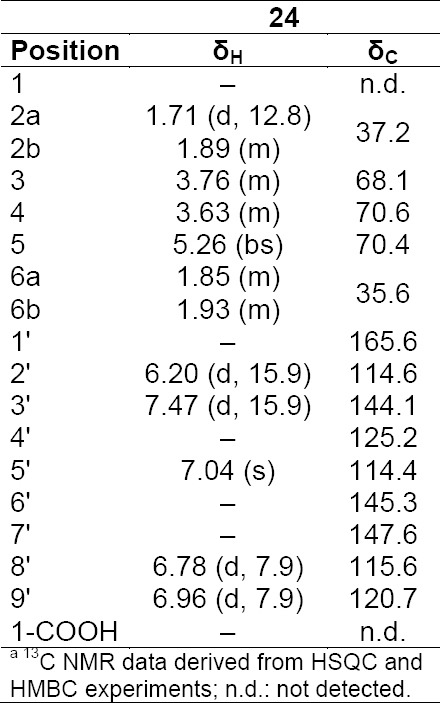
^1^H and ^13^C NMR (500 MHz) of compounds **24** in DMSO-d6

**Tab. 12S:**
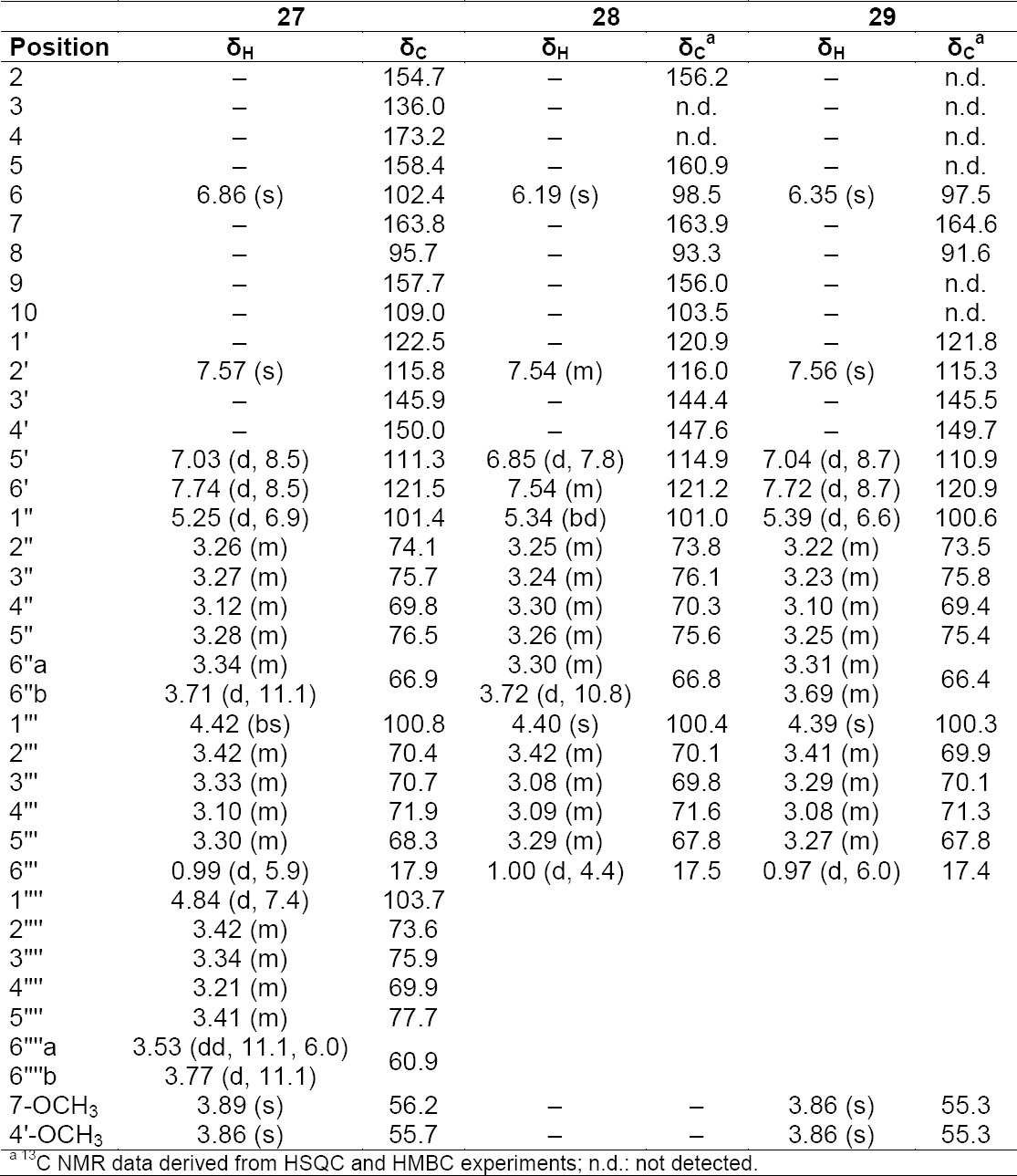
^1^H and ^13^C NMR (500 MHz) of compounds **27–29** in DMSO-d6

## Supporting Information

A list of the 19 active extracts, additional activity profiles for *B. frutescens*, polyamide profiles of *M. splendens*, *C. aff. laxum*, and *E. macrophyllum*, and NMR data of compounds **1**–**8**, **10**, **12**–**17**, and **19**–**29** are available in the online version (Type: PDF, Size: ca. 0.5 MB): http://dx.doi.org/10.3797/scipharm.1410-14
